# Falling through the gaps: how should HIV programmes respond to families that persistently deny treatment to children?

**DOI:** 10.7448/IAS.19.1.20789

**Published:** 2016-05-23

**Authors:** Joanna Busza, Ann Strode, Ethel Dauya, Rashida A Ferrand

**Affiliations:** 1 Department of Population Health, London School of Hygiene and Tropical Medicine, London, UK; 2 School of Law, University of KwaZulu Natal, Pietermaritzburg, South Africa; 3 Biomedical Research and Training Institute, Harare, Zimbabwe; 4 Department of Clinical Research, London School of Hygiene and Tropical Medicine, London, UK

**Keywords:** Zimbabwe, children, antiretroviral treatment, access, human rights, caregivers

## Abstract

**Introduction:**

Children living with HIV rely on adult caregivers for access to HIV testing and care, including clinical monitoring and adherence to treatment. Yet, many caregivers confront barriers to ensuring children's care, including fear of disclosure of the child's or the parents’ HIV status, competing family demands, fluctuating care arrangements and broader structural factors such as entrenched poverty or alternative beliefs about HIV's aetiology and treatment. Thus, many children are “falling through the gaps” because their access to testing and care is mediated by guardians who appear unable or unwilling to facilitate it. These children are likely to suffer treatment failure or death due to their caregivers’ recalcitrance.

**Discussion:**

This Commentary presents three cases from paediatric HIV services in Zimbabwe that highlight the complexities facing health care providers in providing HIV testing and care to children, and discusses the implications as a child's rights issue requiring both legal and programmatic responses. The cases provide examples of how disagreements between family members about appropriate care, conflicts between a child and caregiver and religious objections to medical treatment interrupt children's engagement with HIV services. In all three cases, no social or legal mechanisms were in place for health staff to intervene and prevent “loss to follow up.”

**Conclusions:**

We suggest that conceptualizing this as a child's rights issue may be a useful way to raise the debate and move towards improved treatment access. Our cases reflect policy failure to facilitate access to children's HIV testing and treatment, and are likely to be similar across international settings. We propose sharing experiences and encouraging dialogue between health practitioners and global advocates for children's right to health to raise awareness that children are the bearers of rights even if they lack legal capacity, and that the failure of either the state or their caregiver to facilitate access to care is in fact a rights violation.

## Introduction

The World Health Organization estimates that 740,000 HIV-infected children below the age of 15 were taking antiretroviral treatment in 2014 [1], while in 2013 there were 190,000 deaths in this age group caused by lack of treatment [2]. Children living with HIV generally rely on adult caregivers for access to HIV testing and care, including regular clinical monitoring. Caregivers remain responsible for ensuring adherence to medication until the child is old enough to assume this role [3], yet many confront numerous barriers to managing children's diagnosis and treatment [4–7]. These include fear that a child's HIV diagnosis or treatment will result in disclosure of the child's or the parents’ status, reluctance to provide medication in the absence of adequate food, competing family demands that make it difficult to prioritize a particular child's needs, fluctuating care arrangements and changes in caregivers, long distances to health facilities or arduous requirements for collecting drugs and broader structural factors such as entrenched poverty or alternative beliefs about HIV's aetiology and treatment.

Various strategies have been developed to address these issues [8–10]. In Zimbabwe, the ZENITH trial is testing whether support to caregivers through structured home visits by trained lay health workers (LHWs) delivered alongside routine HIV services at local primary care clinics will improve children's retention in care [4]. Ethical approval for the trial has been granted by the Medical Research Council of Zimbabwe and the London School of Hygiene and Tropical Medicine (UK). Participants were enrolled with informed, written consent by a caregiver and assent by children. Results of the trial will be available later in 2016, but it has become clear that some children are “falling through the gaps” because their access to testing and care is mediated by caregivers. During enrolment, 14 eligible children's caregivers refused to participate because they did not accept the HIV test results and a further 11 children were enrolled but never returned for assessment or treatment. Among study participants, visiting LHWs have confronted varying degrees of caregiver reluctance and inability to maximize children's retention in care. These children are likely to suffer treatment failure or death due to their caregivers’ recalcitrance.

## Discussion

We summarize three cases that highlight the complexities facing health care providers in providing HIV testing and care to children in Zimbabwe and discuss their implications as a child's rights issue, requiring both legal and programmatic responses. All names and identifying characteristics have been changed to protect study participants.

### Case 1: Disagreements between adult family members

Belinda is 13 years old and was diagnosed HIV-positive following routine provider-initiated testing and counselling (PITC). Her mother consented to home visits by an LHW but did not bring her daughter for her initial clinic appointment. A follow-up phone call by study nurses prompted no response, and the LHW and a nurse visited Belinda's home. They spoke with her grandmother, who claimed that as head of the household, it was her right to decide whether the child should access clinical care. She doubted the HIV diagnosis on the basis that Belinda's father had died in a road traffic accident and her mother was “not ill.” Subsequently phone calls were made to Belinda's mother, with no response. Eleven months later, Belinda presented to the clinic complaining of weight loss, night sweats and fever. Although her mother requested treatment, her grandmother continued to disallow it. There were no further clinic visits and her mother claimed Belinda was being treated at a private clinic, although our staff found no record of her when they checked. Further phone calls were ignored and Belinda was deemed lost to follow up (LFTU).

### Case 2: Conflict between child and caregiver

Williams, 15, attended the clinic on his own requesting an HIV test. Because he was under 16, HIV testing required guardian consent. He insisted on having an HIV test, suspecting he was HIV-positive as both parents had died of AIDS-indicative conditions. A compromise was reached whereby the HIV test was performed but the results could be disclosed to him only in the presence of his aunt. The aunt and Williams never returned. At follow-up phone calls, the aunt claimed she had a full-time job and did not have the time to bring the child for care. Follow-up visits were made by an LHW but each visit resulted in the aunt shouting and threatening Williams. The research staff have since learned that Williams has been enrolled at a boarding school 300 km away, and continues to receive no care.

### Case 3: Religious opposition

Angel, an 11-year-old maternal orphan, attended the clinic with her biological father and tested HIV-positive following PITC. When she did not return for her scheduled appointment, home visits were arranged. Angel's father follows an evangelical religion that believes in prophesy and often discourages modern medical treatment. He declared his daughter was “bewitched” according to what the prophet advised and did not require any treatment by the clinic. The clinic staff reported the case to social services but have not heard of any subsequent developments. Angel has been recorded as LFTU.

These cases occurred within a paediatric HIV study able to make intensive efforts to interact with children's caregivers. Larger and more heavily burdened public services are less able to devote time and scarce resources to following up children and addressing complex issues such as those described above, resulting in a significant minority of children living with HIV unable to access HIV testing and/or care. Zimbabwe's National HIV treatment guidelines state that health providers need to act “in the best interest of the child” and specify that a hospital practitioner can overrule guardian refusal of inpatient care, but these stipulations do not address issues that arise at home and lead to missed outpatient appointments. These children are labelled as lost to follow-up, concealing complex issues that underpin their poor uptake and retention in HIV care.

## Conclusions

We suggest that conceptualizing this as a child's rights issue may be a useful way to raise the debate and move towards improved treatment access. Children's rights are a specific sub-set of human rights. Through the Convention on the Rights of the Child (CRC), they have become a core element of international law [11]. Article 24 states that every child has the right to “the enjoyment of the highest attainable standard of health and to facilities for the treatment of illness and rehabilitation of health” [12]. Although it does not expressly deal with HIV testing and treatment, General Comment No. 3 states:

The accessibility of voluntary, confidential HIV counselling and testing services, with due attention to the evolving capacities of the child, is fundamental to the rights and health of children. … Consistent with their obligation under article 24 of the Convention to ensure that no child is deprived of his or her right of access to necessary health services. (General Comment No. 3, 2003)

Our cases involve young children who do not have legal capacity to consent to HIV testing or treatment and require an adult to act on their behalf. They reflect the failure of the legal framework to facilitate access to HIV testing and treatment by ensuring the law specifies who may provide proxy consent, broadening the categories of persons who may assist children or enabling children with sufficient capacity to consent independently.

Countries that have ratified the CRC accept responsibility to ensure that “no child is deprived of his or her right of access to such health care services” [13]. This places a direct obligation on them to ensure laws and policies are in place to operationalize this principle so that programmes give effect to it. In recent years, some countries in Southern Africa have reformed children's laws or adopted HIV-specific laws allowing older children to consent independently to HIV testing. For example, children can consent to HIV testing and medical treatment at 16 in Botswana [14], 12 in Lesotho [15], South Africa [16] and 14 in Namibia [17]. Despite these laws, some providers appear unwilling to go against caregivers’ wishes or are uncertain of how to do so if there is a conflict between the child and the caregiver [18]. In contexts where there are fewer guidelines, weaker infrastructure and inadequate social services, the gap between a new law and subsequent practice may be even wider.

We suggest that there are two ethical and legal questions regarding how governments should take the principles in the CRC forward that are relevant to these and similar situations:

1) *Is this a situation in which the state should intervene through using existing laws relating to abuse, maltreatment and neglect?* Can we classify this as a form of abuse, maltreatment or neglect? At what point is caregivers’ lack of engagement life threatening? 2) *If yes, what form should state intervention take?* Should children be removed to places of safety? Should caregivers’ consent be overridden by, for example, social workers or the heads of health facilities? Should laws be reformed to allow children to consent to testing at a younger age? Should the definition of caregiver be broadened to include a wider range of persons? Is there a role for social workers to collaborate with health care providers?

To start answering these questions, we propose a range of strategies. First, legislative reform should enable older children to consent to treatment independently and facilitate a range of other persons to provide consent in certain circumstances. Second, dialogue with key stakeholders should discuss circumstances in which the state ought to use child protection legislation to ensure a child's health rights are protected. Third, community-based programmes addressing stigma and myths on HIV should be strengthened. Finally, we suggest broadening this debate through sharing experiences and encouraging dialogue between health practitioners and global advocates for children's right to health. We welcome the recent call by the Coalition for Children Affected by AIDS for greater inclusion of children's issues in structural interventions [19]. Framing these experiences as human rights issues can raise awareness of the failure of health professionals to recognize that children are the bearers of rights even if they lack legal capacity, and that the failure of either the state or their caregiver to facilitate access to care is in fact an actionable rights violation.
